# Detection of Blood Stains

**Published:** 1871-06

**Authors:** 


### Detection of Blood Stains.

Iodine of potassium dissolves traces of blood, even from clothing which has been thoroughly washed, but hæmin crystals cannot be obtained from the solution.

Gunning has discovered, in the acetate of zinc, a reagent that precipitates the slightest traces of the coloring matter of blood from solutions, even where the liquids are so dilute as to be colorless. Blood washed from the hands in a pail of water can readily be detected in this way. The flocculent precipitate, thrown down by the acetate of zinc, must be washed by decantation, and finally collected on a watch glass, and allowed to dry, when the microscope will readily reveal hæmin crystals, if any blood be present. This test has been repeatedly tried with entire success.

